# Mucoadhesive Gels Designed for the Controlled Release of Chlorhexidine in the Oral Cavity

**DOI:** 10.3390/pharmaceutics3040665

**Published:** 2011-09-27

**Authors:** Adamo Fini, Valentina Bergamante, Gian Carlo Ceschel

**Affiliations:** 1 Department SMETEC, University of Bologna, Bologna, 40127, Italy; E-Mail: valentina.bergamante@unibo.it; 2 Monteresearch, Bollate, Milano, 20021, Italy; E-Mail: giancarlo.ceschel@monteresearch.it

**Keywords:** chlorhexidine, mucoadhesive gels, release/permeation

## Abstract

This study describes the *in vitro/ex vivo* buccal release of chlorhexidine (CHX) from nine mucoadhesive aqueous gels, as well as their physicochemical and mucoadhesive properties: CHX was present at a constant 1% w/v concentration in the chemical form of digluconate salt. The mucoadhesive/gel forming materials were carboxymethyl- (CMC), hydroxypropylmethyl- (HPMC) and hydroxypropyl- (HPC) cellulose, alone (3% w/w) or in binary mixtures (5% w/w); gels were tested for their mucoadhesion using the mucin method at 1, 2 and 3% w/w concentrations. CHX release from different formulations was assessed using a USP method and newly developed apparatus, combining release/permeation process in which porcine mucosa was placed in a Franz cell. The combination of HPMC or HPC with CMC showed slower drug release when compared to each of the individual polymers. All the systems proved suitable for CHX buccal delivery, being able to guarantee both prolonged release and reduced transmucosal permeation. Gels were compared for the release of previously studied tablets that contained Carbopol and HPMC, alone or in mixture. An accurate selection and combination of the materials allow the design of different pharmaceutical forms suitable for different purposes, by simply modifying the formulation compositions.

## 1. Introduction

Many pharmaceutical researchers have focused on formulations enabling drug targeting and prolongation of the therapeutic effect in the oral cavity; protection of the drug from degradation in the adverse biologic environment was an additional focus. These requirements can be fulfilled by a suitable mucoadhesive formulation inside the buccal cavity: conventional dosage forms, in fact, maintain their effects in this cavity for a significant period of time with difficulty, because they are very easily removed by salivation, temperature, tongue movement, and swallowing. Moreover, the buccal mucosa is a suitable region for bioadhesive systems because of its smooth and relatively immobile surface and direct accessibility: as a consequence, suitable mucoadhesive formulations represent an alternative to conventional uncontrolled drug delivery forms [1].

Mucoadhesive dosage forms, including tablets, gels, and films, have been extensively developed for the treatment of oral cavity diseases [2]. Important limitations, concerning the size of mucoadhesive tablets, whose thickness must be limited to about 1 mm and which must be soft enough to be acceptable to patients and not cause irritation [3], suggest the use of mucoadhesive films or gels that represent an alternative to solid forms, since they can offer a larger and softer surface area of release in the buccal area for an extended period of time, due to their viscosity [4,5] and add compliance in terms of flexibility and comfort. Adhesive films and laminated patches, used as buccal delivery systems, present some disadvantages associated with the solvent casting method, however, such as environmental concerns, long processing times, and high costs [6] and appear more useful to deliver drugs directly to a mucosal membrane.

Although a limitation of gel formulations lies in their inability to deliver a measured dose of drug to the site, gels have some advantages over other formulation types, such as relatively faster release of the incorporated drug and easy preparation; easier administration and higher biocompatibility and mucoadhesivity, allow adhesion to the mucosa in the dental pocket and rapid elimination through normal catabolic pathways and decrease the risk of irritative or allergic host reactions at the application site. Semisolid mucoadhesive dosage forms, such as gels (or ointments), that represent excellent formulations for several routes of administration, such as topical, vaginal and rectal, may therefore be considered efficient as drug delivery systems in the buccal cavity, covered by a mucus layer: mucoadhesive gels are easily dispersed throughout the oral mucosa, even if drug dosing from these pharmaceutical forms may not be as accurate as from tablets, patches, or films. Many adhesive gels are employed in the local delivery of chlorhexidine (CHX) for the treatment of periodontitis or various inflammatory and infectious diseases of the mucosa and teeth or the release of antimicrobial agents, with high efficacy and patient acceptability [7,8].

The purpose of this study was to develop formulations and evaluate *in vitro* performances of mucoadhesive gels for buccal release using chlorhexidine, displaying prolonged effects, while minimizing transmucosal permeation, and avoiding toxicity problems. The release of CHX from the gel systems was compared to the release from previously studied mucoadhesive tablets [9].

As gel forming agents in this paper we considered ionic (carboxymethyl cellulose—CMC) and/or non ionic (hydroxypropyl- and hydroxypropylmethyl- cellulose) polymers, testing a number of combinations, capable of generating synergism towards mucoadhesion and release control, while minimizing absorption.

As a model drug in these formulations, we tested CHX. In addition to a number of effective drugs for local therapy of the oral cavity, this drug is successfully employed not only for its activity against a wide number of microbial species, but also as an adjunctive supplement in oral candidosis, since it reduces the adhesion of *Candida albicans* to oral mucosal cells [10]. However, its prolonged use, e.g. in mouthwash, not only has a disagreeable taste, but can also lead to the formation of brown spots on the surface of the teeth: the amount of staining being dependent on the mode of application and concentration; cosmetic problems associated with intraoral staining are a factor which decreases patient compliance [11]. As a consequence, in order to use this agent with limited side effects, it is important to achieve lower concentrations at therapeutical level for prolonged periods, mucoadhesive gel formulations therefore being indicated as suitable forms for CHX delivery and treatment of disorders of the oral cavity.

## 2. Results and Discussion

### 2.1. Gel Forming Polymers

Polymers displaying mucoadhesive properties are capable of hydrogen-bond formation, possess swelling/water load properties and sufficient flexibility for entanglement with mucus: the selected cellulose derivatives (hydroxypropyl methylcellulose, hydroxypropyl cellulose, carboxymethyl cellulose) fulfill all these requirements [12] and were used to prepare the mucoadhesive gels for the release of CHX, alone or in the mixtures.

Hydroxypropyl methylcellulose (HPMC) is a mixed alkyl/hydroxyalkyl cellulose ether containing methoxyl and hydroxypropyl groups, commonly used in hydrophilic matrix drug delivery systems and frequently as the gel base to provide sustained release. It is available in a wide range of molecular weights and is classified by the viscosities of the 2% (w/w) aqueous solution The polymer is an important water-soluble excipient also used for the preparation of oral controlled drug delivery systems. It displays thickening property, pH stability, water retention and adhesion power [13] also together with other polymers [4]. Hydroxypropyl cellulose (HPC), likewise HPMC, is a non-ionic water soluble cellulose ether with a remarkable combination of properties. It has the thickening and stabilizing properties characteristic of other water-soluble cellulose polymers. While these two polymers are insensitive to electrolytes, carboxymethyl cellulose (CMC) is affected both by ionic strength and pH; it is a well-known mucoadhesive, ionizable, semi synthetic water-soluble polymer, in which -CH_2_COOH groups are substituted on the glucose units of the cellulose chain through an ether linkage: it has been used in the form of sodium salt [14].

The polymers were used to form the gel formulation alone at 3% w/w or in mixture (3 + 2)% w/w, according to Table 1.

The nine gels were prepared in a very simple manner, dissolving the polymer or the binary mixture in water under stirring and adding the CHX digluconate solution in order to achieve the same CHX concentration (1% w/w): the system is left to gel and used after 24 h.

The gelling agents behave differently when in water. The recommended method to prepare HPMC aqueous solutions is to first thoroughly disperse and hydrate the powder in a portion of hot water (about one third of the total volume), heated above 90 °C with vigorous stirring to prevent lumping; complete dissolution is then accomplished by adding the remaining portion as cold water to lower the temperature of the dispersion. As the temperature is lowered, HPMC becomes water soluble, the solution resulting in increased viscosity (“hot/cold” technique) [15]: in contrast, HPC is known to precipitate and become insoluble at 40–45 °C. When hydrated and dispersed, the polymers undergo a high degree of entanglements or association, which alters the viscosity of the dispersing medium. Attention to the behavior of these polymers was paid in the preparation of the final aqueous gels that displayed the consistency of a soft paste. The presence of the polymers does not affect the solubility of the drug in the gels: in all the cases the saturation concentration of the drug in the nine gels ranged from 18 g/100 mL (gel 2) to 42 g/100 mL (gel 5), well above the value here considered. Also the experimental conditions used in the present work (buffer pH = 7.4) are unable to greatly modify CHX solubility: CHX is a strong base and, at physiologic pH, is in the form of a di-cation, with two positive charges distributed over the nitrogen atoms of the molecule.

All gel systems were evaluated for their mucoadhesion and CHX release.

### 2.2. Mucoadhesion Assay

The present assay is based on the idea that chemical interactions and entanglements between the formulation components and glycoproteins in mucus cause a rheological synergism [16]. To evaluate mucoadhesion of the nine gels, a test with commercial mucin (the main component of mucus) was carried out by measuring the change in viscosity of the mucin/formulation association at increasing concentrations of mucin. This type of measurement is frequently used, even though the results obtained are often difficult to interpret and can vary considerably, depending on a number of experimental parameters, such as the concentration and the ion-sensitivity of the polymer, the quantity of ions present, the mucin type and instrumental factors.

In the presence of interactions, a rheological synergism could be observed that represents a more than additive growth of the mixture viscosity that occurs when mucoadhesive polymers are mixed with mucin dispersions, depending on the interactions between the chains of the two macromolecular species. In other words, the term [*η*_mixture_ − (*η*_polymer_ + *η*_mucin_)] > 0 represents the extra contribution to viscosity of the mucin-polymer interaction, with reference to the value expected on the basis of a simple additivity of the polymer and mucin contributions.

The viscosity values of all the systems examined are higher than the sum of the corresponding values of the separate components at all the mucin concentrations investigated: as a consequence, all the formulations examined showed a positive value of the term to different extents. The samples containing CMC showed an important synergism and were therefore employed for further release/permeation tests. This was partly expected since in the large classes of hydrophilic polymers, those containing the carboxylic group exhibit the best mucoadhesive properties [12].

**Figure 1.**
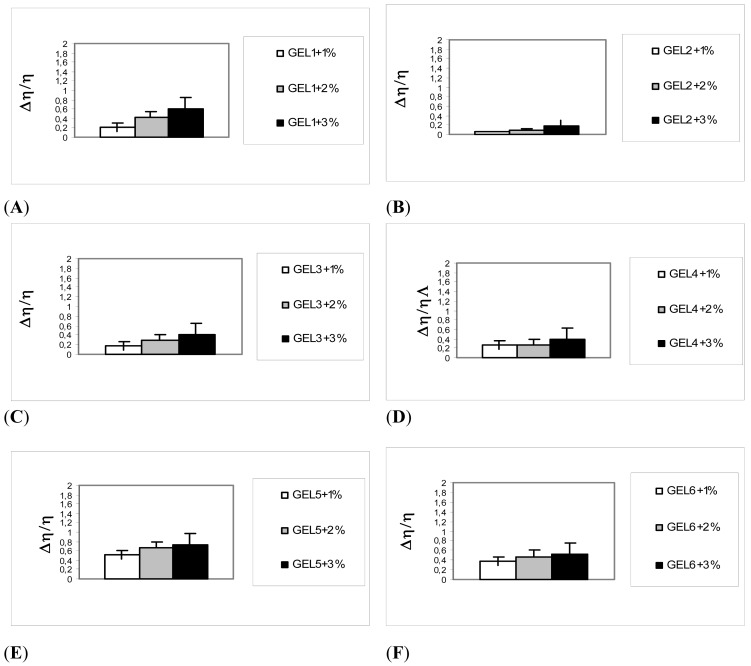

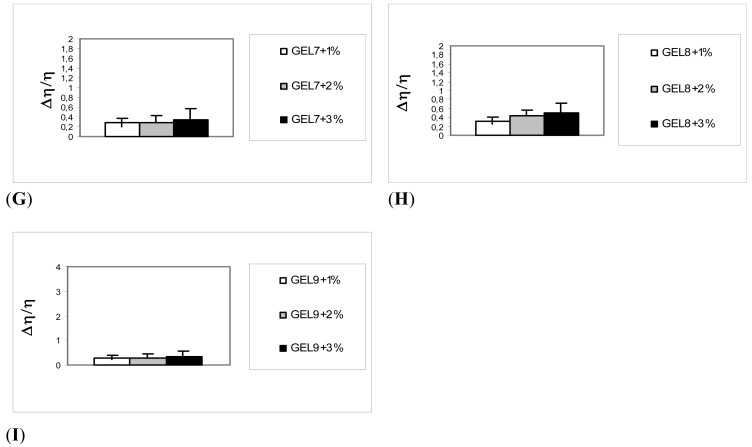
Rheological test with mucin. Gels 1, 4, 5, 6 and 8 contain carboxymethyl cellulose (CMC) in (**A**)–(**I**).

Figure 1 shows the results for the nine systems; positive interactions were displayed to an important extent particularly for the systems 1, 4, 5, 6 and 8, as reflected in an increase in the residence time on the mucosa as a result of binding to the mucus layer coating the buccal epithelium. These gels were considered for further tests.

### 2.3. Release Tests

**Figure 2.**
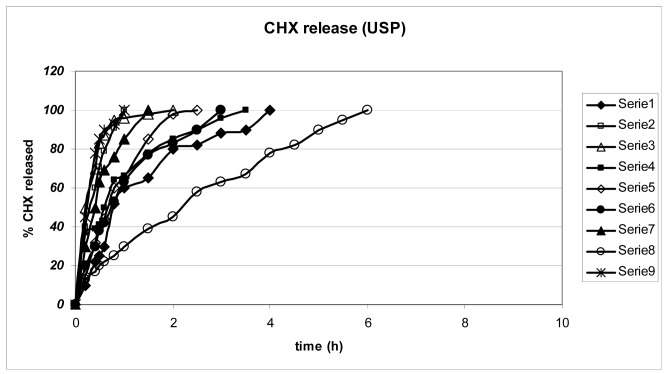
Release profiles according to the USP method. The numbers are as in Table 1.

**Figure 3.**
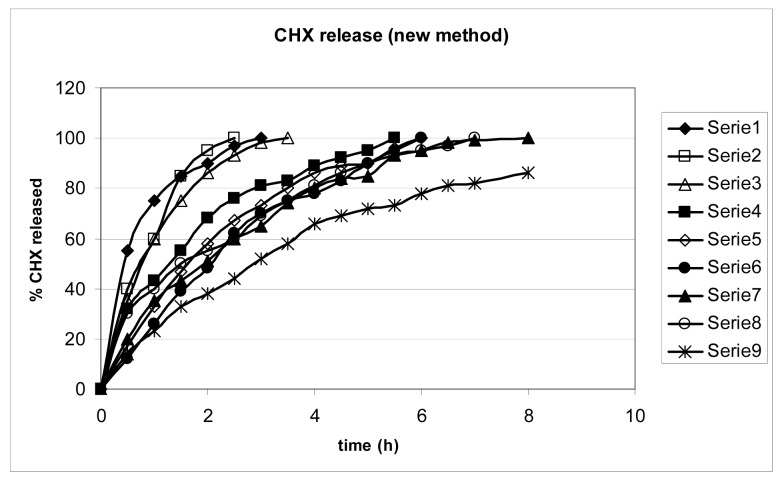
Release profiles according to the new method: the numbers are as in Table 1.

To test the ability of the gels to release CHX, we employed the USP 26 paddle standard method (Figure 2) and compared the results with those obtained using a new apparatus (Figure 3), previously described to mimic the movements of the mouth [9].

The gels completely release CHX, though at different rates, according to the nature of the gel-forming polymer as well as its concentration (Figure 2): gel 8 was able to prolong and control CHX release for more than 4 h; while the release from gels 2, 3, 6 and 7, which are those rich in HPMC, was too fast and poorly reproducible. Gels 1, 4, 5, 6 and 8, which are those richer in CMC, prolong the CHX release.

It also appears that when the gels are prepared from a single component, they release CHX in a comparable way, without any control, and release is complete in about 2 h; formulations where polymers are in binary mixtures are associated with a decrease in the drug-release rate: the CMC/HPC system in Gel 8 exerts the best control on CHX release that is complete only after 4 h.

The two methods do not offer comparable results: the new one appears to produce more regular profiles and a more prolonged release: the sequence of the release rate appears comparable in the two cases.

Affinity of the cationic CHX for the negatively charged CMC is expected and binding of the functional groups of the polymer and the ionic sites of CHX can result in delayed release of the drug from the polymeric matrix: depending on the concentration of the drug, CHX positive charges can be almost completely saturated by CMC carboxyl groups

The ionic interaction between CHX and a bioadhesive polycarboxylate was demonstrated by the shift in the strongest spectrum band of CHX, when protonated by the carboxylic groups of the polymer [17].

The same effect on the release was observed for CHX also in the presence of different polymers carrying carboxylic groups, such as polyacrylic acid [9,10] or alginate [18], suggesting a common mechanism for the control of CHX release from systems containing acidic polymers: the formation of a complex can be responsible for a longer CHX retention in the gel.

The occurrence of anionic interactions is not uniquely responsible for drug retention in the polymer gel; probably viscosity of the hydrated gel plays a role in modulating the diffusion of CHX through the polymer matrix. As a consequence the association of an ionic with a non-ionic polymer in the gel systems provides a better control of the release: CMC slows down and HPMC or HPMC, depending on their ability to control the matrix viscosity, favor the CHX release. Figure 2 suggests that the optimum association is that of Gel 8 (CMC/HPC = 2/3) to obtain a sustained release of CHX from the gel.

### 2.4. Release/Ex Vivo Permeation Test

**Figure 4.**
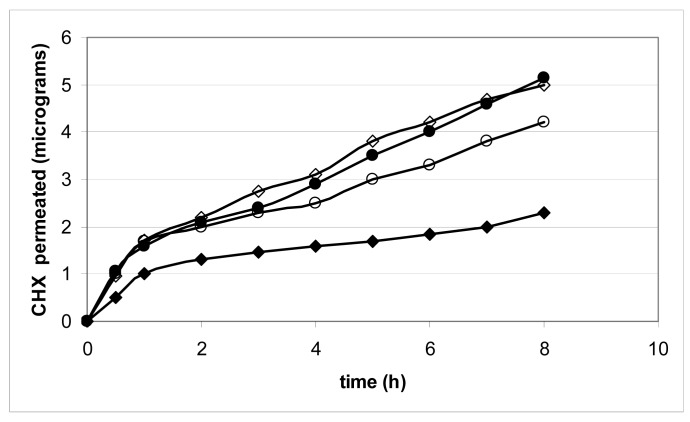
Permeation profile of CHX from Gels: 1 ◆ 5 ◊ 6 ● 8 ○, using a modified Franz cell.

**Figure 5.**
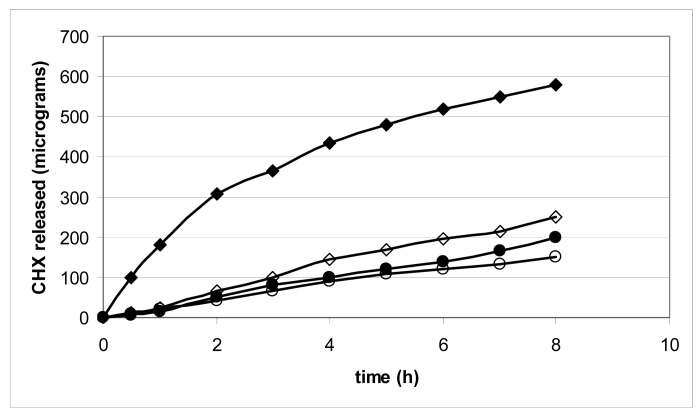
Release profile of CHX from Gels: 1 ◆ 5 ◊ 6 ● 8 ○ using a modified Franz cell.

Figures 4 and 5 show important results of the release/permeation test. The test was carried out using gels 1, 5, 6 and 8, placed on the porcine mucosa in a modified Franz cell to evaluate both CHX release and permeation across the porcine mucosa.

Since most intraoral surfaces, as well as the bacterial cell wall, are negatively charged, by virtue of its positive charge, CHX is well distributed in the oral cavity and exerts its bacteriostatic and bactericidal effects. The di-cationic nature makes CHX interactive with anions and promotes its efficacy, but also prevents side effects. The cationic nature of CHX minimizes absorption through skin and mucosae including that of the gastrointestinal tract.

Figure 4 shows that CHX permeates very poorly across the mucosa during the release study; this is rather important since the site of application can also represents a pathway of absorption.

The nature of the vehicle (aqueous gel) and of the drug (a charged molecule) makes the escape of CHX towards the dissolution medium more probable rather than towards the mucosa surface. The profiles are linear and the difference in the slopes can be attributed to differences in the gel formulation; the steady state fluxes of the system examined are in the order: 6 > 5 > 8 > 1.

With the exception of Gel 1, the other three samples showed an almost parallel and constant release after 8 h, sufficient to guarantee CHX activity (Figure 5). The amount that Gel 1 released after 8 h, which contains only CMC, is more than double that of the other gels examined. However, it is far from being complete: in this test the mechanical action present in the two release tests used did not operate, as the releasing system and the dissolution fluid enter into contact in different ways.

Table 2 shows the values obtained for flux (J) and permeability coefficient (P) for formulations 1, 5, 6 and 8. The values reported in the literature, for permeability across the buccal mucosa for different molecules, range from a lower limit of 2.2 × 10^−9^ cm/s for dextran 4000 across rabbit buccal membrane to an upper limit of 1.5 × 10^−5^ cm/s for both benzylamine and amphetamine across rabbit and dog buccal mucosa, respectively [19,20].

These values demonstrate the presence of a permeability barrier of the mucosa, which is mostly imposed by the external epithelium acting as a protective layer and as a barrier to the entry of foreign material.

The values found in the present work for CHX from the examined gels are almost in the middle of this range and confirm the difficulty for CHX, a relatively small but charged molecule, to cross the buccal membrane, but also due to the control exerted by the formulation on its permeation. Log*P* values are also reported for CHX and its salts [21,22]: the free base has an octanol/water Log*P* value 0.754, demonstrating a sufficient hydrophobicity to guarantee the crossing of lipophilic membranes. The value drops to 0.047 for the diacetate and 0.037 for the digluconate salt, suggesting a residual even though low hydrophobicity of the compounds, able to partition, e.g. as ion-pairs. The low Log*P* values for the salts confirm the results of the low permeation observed across the porcine mucosa.

From all these results it emerged that a constant release is common to all the mucoadhesive gels examined. Gel 5, however, can be proposed as useful for daily disinfection of the oral cavity, due to its good mucoadhesion that ensures stability of the applied formulation despite the considerable stresses existing inside the oral cavity, and a low degree of absorption across the model membrane.

### 2.5. Comparison with Mucoadhesive Tablets

In a previous study [9] we examined the CHX release from mucoadhesive tablets, prepared by Carbopol and HPMC: this form proved suitable to control CHX release and limit its permeation.

Comparison of the release profiles of the two types of formulations with a very similar composition (both containing HPMC and an anionic polymer: CMC for the gels and Carbopol for the tablets) reveal differences that can be interpreted as follows.

- CMC, in the gels, allows a more rapid release than Carbopol in the tablets that could be useful in acute situations, when a prompt release and relatively high anti-infective agent concentration is needed.
- Carbopol, which offers better control of the release of CHX in the tablets, by means of a higher charge density on its chains, can be suggested for therapies that require prolonged treatment or prevention of buccal infections, covering, e.g. a whole night.
- Tablet manufacture requires a more complex technology than gel preparation: gels can be applied to the buccal mucosa in a simpler way compared to the tablet; moreover they can be inserted in the buccal pocket by means of a periodontal syringe to treat periodontitis: mucoadhesion ensures the necessary retention.
- Finally it must be considered that these different goals were achieved using two different chemical forms of CHX, *i.e.*, the diacetate, as a solid salt inside the tablets, and the digluconate salt, which is present as a solute inside the gels. This fact introduces the dissolution rate as an important variable, whose control represents an additional and useful parameter to drive the release of CHX in different situations, together with common, widely used and inexpensive cellulose polymers.

## 3. Experimental Section

### 3.1. Materials

Chlorhexidine digluconate (CHX) 20% w/v aqueous solution and mucin type II (crude porcine gastric) were purchased from Sigma Aldrich (Milan, Italy). The following pharmaceutical grade polymers: Hydroxypropylmethyl cellulose—HPMC (Methocel K100 Premium LVCR EP: 22.8% methoxyl content, 8.7% hydroxypropyl content, and 107 centipoise (cPs) apparent viscosity as a 2% aqueous solution], Hydroxy propyl cellulose—HPC (Klucel, 60–100 μm, MW 1150 kDa), sodium carboxymethyl cellulose—CMC (viscosity grade: 500–2500 mPas were supplied by Eigenmann and Veronelli S.p.A (Milan, Italy). Artificial saliva was prepared according to Preetha and Banerjee [23]. Bidistilled water was used in all formulations. Other chemicals and reagents were analytical or high-performance liquid chromatography grade.

### 3.2. Methods

#### Preparation of Mucoadhesive Gels

Mucoadhesive polymers, in the range 3% (alone—Gel 1, 2 and 3) or (3 + 2)% w/w (in mixture—Gel 4–9), according to Table 1, were dissolved in warm water (60–65 °C) under stirring: CHX solution was added up to 1% w/v final concentration and the systems were left to jellify and used after 24 h.

#### Solubility Determination

The CHX equilibrium solubility in various vehicles was measured at 25 °C. Gel prepared as described were centrifuged at 4000 rpm. The surnatant was recovered, filtered (0.45 μm nylon filter, MSF, Dublin, USA) and essayed for the content in CHX by HPLC method (see below). All experiments were carried out in triplicate.

#### Mucoadhesion Assay of the Gel Systems

A viscometric method was used to evaluate mucin-polymer interaction: this method is based on the evaluation of the *rheological synergism* existing between the mucoadhesive polymer and mucin [24,25].

Viscosity was measured at 37 ± 1 °C, by means of a rotational or Ostwald viscosimeter (*η*_polymer_) for mucin dispersions (1, 2 and 3% w/w) in the absence (*η*_mucin_) and presence of the selected polymer solution (*η*_mixture_) and for polymer solutions (*η*_polymer_, in the absence of mucin). The viscosity component of bioadhesion (rheological synergism) was calculated from the following equation: Δ*η* = [*η*_mixture_ – (*η*_polymer_ + *η*_mucin_)]. Figure 1 shows the results in term of the Δ*η*/*η*_polymer_ ratios.

All experiments were carried out in triplicate.

#### In Vitro Release Test Using a USP Paddle Apparatus

Gels were evaluated for *in vitro* CHX release in 900 mL of pH 7.4 phosphate buffer, using the USP 26/NF paddle method, at 50 rpm and 37 ± 0.2 °C. Each gel was placed in a separate pan present at the bottom of the dissolutor. At regular time intervals, 20 μL of solution were withdrawn and assayed for CHX, using the HPLC method. All experiments were carried out in triplicate. CHX release from the prepared gels was measured also using an unofficial method for comparison. The apparatus was designed to better simulate the action of saliva in the mouth on the gel surface. It consists of a pump that extracts a pH 7.4 buffer solution from a thermostated beaker and drips the solution on the gel from a distance of 2 cm (flux: 1 mL/min) and was previously described in details [9]. Release profiles are shown in Figures 2 and 3. The data are the mean of three independent tests.

#### Tissue Preparation

Porcine buccal mucosa, with a fair amount of underlying connective tissue, was surgically removed from the oral cavity of a freshly killed male pig obtained, on each study day, from a local slaughter house (CLAI, Imola, Italy). The buccal mucosa was placed in ice-cold phosphate buffer 0.15 M. The connective tissue of the mucosa was carefully removed using fine-point forceps and surgical scissors. The cleaned buccal mucosa membrane was then placed in ice-cold pH 7.4 phosphate buffer 0.06 M until it was mounted in the diffusion cells. The thickness of the porcine buccal mucosa was assessed by means of an electronic caliper: the main value found for the present sample was 1.0 ± 0.1 mm.

#### In Vitro Release and Permeation Test

This test was carried out using a previously modified Franz method [26]. The inferior compartment, filled with 4.8 mL of artificial saliva (pH 7.0), simulated the oral cavity, while the superior compartment, filled with 3 mL of pH 7.4 phosphate buffer, simulated the blood circulation. A porcine buccal mucosa was clamped with the external surface turned towards the inferior compartment. The gel adhered to the external mucosal surface into the inferior compartment, where the solution was continuously stirred at 600 rpm using a Teflon coated magnetic stirrer to simulate the mechanical movements of the mouth. The amount of CHX released in the simulated oral cavity layer was determined by removing 20 μL aliquots from the inferior compartment every 30 min, while the amount of drug that permeated through the porcine buccal mucosa was determined by removing the total amount from the superior compartment at 0.5, 1, 2, 3, 4, 5, 6, 7 and 8 h. Samples thus obtained were transferred to volumetric flasks and stored in a refrigerator until they were analyzed by HPLC.

Permeation profiles were constructed plotting the cumulative mass of diffusant, μg, versus time: after a short period of time (lag time), the graph approaches linearity. The steady-state flux *J* was obtained dividing the slope of the linear portion (dm/dt) to the area surface of the porcine buccal mucosa (0.6 cm^2^).

Permeability coefficients (*P*) were calculated as the ratio of the flux *J* to the CHX concentration, that is the concentration of CHX in the releasing gel (1 g% w/v), assuming that all the drug is present as solute. It was also assumed that the drug concentration in the receiver compartment is negligible compared to that in the donor compartment.

The *J* (μg cm^−2^ h^−1^) and *P* (cm s^−1^) values obtained for the gels 1, 5, 6 and 8 are shown in Table 2.

All experiments were carried out in triplicate: data are reported in Figure 3.

#### Spectrophotometer UV/VIS Analysis

CHX was determined using a Spectrophotometer device (UV/VIS Spectrophotometer model Jasco V-530). CHX was analyzed spectrophotometrically at 255 nm using a Shimadzu UV-160A spectrophotometer. The method gave a linear response over a concentration range of 1–20 μmol/mL.

#### HPLC Analysis

CHX was determined using a HPLC apparatus (Model 305, Gilson) equipped with a UV detector set at 239 nm (model Spectra 200, Spectra-Physics). A Nova-Pak C18 (150 × 3.9 mm, 4 mm, Waters) column was used. Elution was carried out at room temperature with a mobile phase consisting of phosphate buffer pH 3 (50%) and acetonitrile (50%); the injecting volume was 20 μL. The flow rate was 1.0 mL/min. In these conditions the retention time of CHX is 5.00 min. A calibration curve was prepared using solution at concentrations ranging from 1 to 20 μmol/mL. In this range the method gave a linear response (*r* = 0.998).

#### Data Analysis

To analyze the release mechanisms of chlorhexidine from the mucoadhesive layer facing the mucosa and to clarify the release mechanism, the release kinetic parameters were calculated using the following equations:

Eq. 1 $$M_{\text{t}}/M_{\infty }=kt^{\text{n}}$$

Eq. 2 $$\log(M_{\text{t}}/M_{\infty })=\log k+n\log t$$

where M_t_/M_∞_ is the fraction of drug released at time *t*, k is a characteristic constant of the formulation and n is indicative of release order kinetic.

From the plot of log (*M*_t_/*M*_∞_) *vs* log *t*, the kinetic parameters, n and k, could be calculated: the slope of the line is n while ln *k* is the intercept on y axis (Table 2).

## 4. Conclusions

- Gels containing cellulose derivative polymers (CMC, HPC, HPMC) display ability of interacting with mucin that can be interpreted as binding to the mucus layer coating the buccal epithelium and increased residence time on the mucosa.
- Gels release CHX at different yields and rates as a function of the nature of the polymer and their association: CMC slows the release of CHX because of electrostatic interactions between the polymer anion and the drug anion.
- The association CMC and HPC in the weight ratio 2/3 allows the preparation of a gel that better sustains the release of CHX from the gel and can be proposed as a formulation for daily disinfection of the oral cavity.
- CMC in gels allows a more rapid CHX release than Carbopol in tablets, which could be useful in acute situations; CHX tablets can be suggested for therapies that require prolonged treatment or prevention of buccal infections.

## Figures and Tables

**Table 1.** Weight % composition of the nine gels; chlorhexidine (CHX) is present at 1% w/w.

Components (weight %)	GEL 1	GEL 2	GEL 3	GEL 4	GEL 5	GEL 6	GEL 7	GEL 8	GEL 9
Carboxymethyl Cellulose (CMC)	3	/	/	3	3	2	/	2	/
Hydroxypropylmethyl Cellulose (HPMC)	/	3	/	2	/	3	3	/	2
Hydroxypropyl Cellulose (HPC)	/	/	3	/	2	/	2	3	3

**Table 2.** Release (*N* and *k*) and permeation (*J* and *P*) parameters: the values, obtained using the new method, are in parentheses.

	GEL 1	GEL 2	GEL 3	GEL 4	GEL 5	GEL 6	GEL 7	GEL 8	GEL 9
*N*	0.75 (0.84)	0.59 (0.50)	0.60 (0.54)	0.48 (0.48)	0.69 (0.74)	0.70 (0.80)	0.58 (0.59)	0.60 (0.50)	0.55 (0.66)
*k* (% release/h)	43.6	57.4	57.0	41.5	60.8	60.7	89.4	30.8	100
*J* (μg/cm^2^.h)	0.27				0.80	0.93		0.60	
*P* (cm/s)	7.5× 10^−9^				22.2 × 10^−9^	25.8 × 10^−9^		16.7 × 10^−9^	
